# Analyzing the Temperature Dependence of Titania Photocatalysis: Kinetic Competition between Water Oxidation Catalysis and Back Electron–Hole Recombination

**DOI:** 10.1021/acscatal.4c03685

**Published:** 2024-10-24

**Authors:** Yohei Cho, Tianhao He, Benjamin Moss, Daniele Benetti, Caiwu Liang, Lei Tian, Lucy Jessica F. Hart, Anna A. Wilson, Yu Taniguchi, Junyi Cui, Mengya Yang, Salvador Eslava, Akira Yamaguchi, Masahiro Miyauchi, James R. Durrant

**Affiliations:** † Department of Chemistry and Centre for Processable Electronics, Imperial College London, London W12 0BZ, United Kingdom; ‡ Department of Materials Science and Engineering, School of Materials and Chemical Technology, Tokyo Institute of Technology, 2-12-1 Ookayama, Meguro-ku, Tokyo 152-8552, Japan; § Graduate School of Advanced Science and Technology, Japan Advanced Institute of Science and Technology, 1-1 Asahidai, Nomi, Ishikawa 923-1292, Japan; ∥ Department of Chemical Engineering and Centre for Processable Electronics, Imperial College London, London SW7 2AZ, United Kingdom

**Keywords:** photoanode, temperature, TiO_2_, water oxidation, back electron–hole recombination

## Abstract

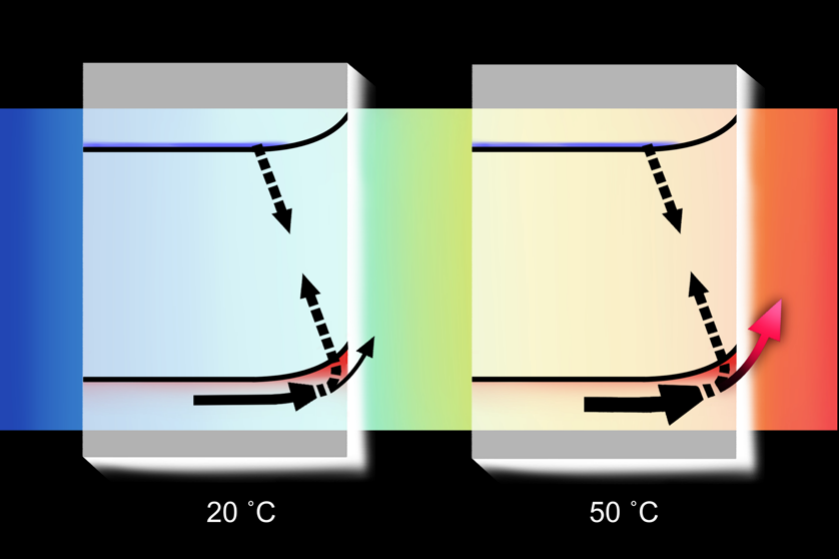

This study examines the kinetic origins of the temperature dependence of photoelectrochemical water oxidation on nanostructured titania photoanodes. We observe that the photocurrent is enhanced at 50 °C relative to 20 °C, with this enhancement being most pronounced (by up to 70%) at low anodic potentials (<+0.6 V vs RHE). Over this low potential range, the photocurrent magnitude is largely determined by kinetic competition between water oxidation catalysis (WOC) and recombination between surface holes and bulk electrons (back electron–hole recombination, BER). We quantify the BER process by transient photocurrent analyses under pulsed irradiation. Remarkably, we find that the kinetics of BER (∼90 ms half-time) are independent of temperature. In contrast, the kinetics of WOC, determined from the analysis of the photoinduced absorption of accumulated surface holes, are found to accelerate up to 2-fold at 50 °C relative to 20 °C. We conclude that the enhanced photocurrent densities observed in the low-applied potential region result primarily from the accelerated WOC, reducing losses due to the competing BER pathway. At higher applied potentials (>+0.6 V vs RHE), a smaller (∼10%) enhancement in photocurrent density is observed at 50 °C relative to 20 °C. Photoinduced absorption studies, correlated with studies using triethanolamine as a hole scavenger, indicate that this more modest enhancement at anodic potentials primarily results from an enhanced charge separation efficiency. We conclude by discussing the implications of these results for the practical application of photoanodic WOC under solar irradiation, influenced by these temperature-independent and -dependent underlying kinetic processes.

## Introduction

There is increasing interest in photocatalytic water splitting for the synthesis of green hydrogen, initially motivated by the pioneering work of Fujishima and Honda on titania photoanodes.^1^ A key consideration for the practical development of these photocatalytic devices is how device efficiency changes with variations in reaction temperature.^2−4^ Several studies have highlighted enhanced photocatalytic performance as the reaction temperature rises under solar irradiation.^5−9^ However, the origin of these temperature-induced changes in photocatalytic performance, particularly the temperature dependence of the underlying charge and catalysis kinetics, has received limited study to date. Examining the temperature dependence of these kinetics can provide insights not only into how to optimize the photocatalytic performance at different operating temperatures but also into understanding the activation barriers associated with the different underlying kinetic processes.

Water oxidation catalysis (WOC) on metal oxide photoanodes requires the surface accumulation of oxidizing species, typically valence band holes, to drive the slow kinetics of WOC. Efficient performance requires both the efficient transport of holes to the surface, avoiding bulk recombination (often quantified as the “charge separation efficiency”, η_sep_), and the efficient reaction of these surface holes with water, avoiding “surface” or “back electron–hole” recombination (BER) (often quantified as the “charge transfer efficiency”, η_tr_).^10,11^ Both recombination losses can be suppressed by the space charge layer formed under the applied anodic potential. In particular, kinetic competition between BER and WOC has been shown to be the key determinant of the applied potential required for the onset of WOC for several metal oxide photoanodes.^12−14^ However, analyses of this kinetic competition as a function of reaction temperature and how this impacts the photocatalytic efficiency have been absent from the literature to date. This competition is particularly critical at low applied potentials, where BER competes with WOC.

In this study, we examine the temperature dependence of the charge separation, recombination, and WOC kinetics in high-quantum efficiency, nanostructured titania photoanodes, thereby determining their impact on the overall temperature dependence of photocatalytic performance. Most studies of the competition between BER and WOC have been conducted using impedance-based analysis.^15,16^ While these studies can be conducted operando with high sensitivity, they are restricted to monitoring the kinetics of extracted electrons. Determining the kinetics of the surface holes driving WOC requires the use of equivalent circuit models, complicating the unambiguous determination of the underlying hole dynamics (see, for example, ref (10)). In order to avoid some of these ambiguities, herein, we employ both photocurrent transients to monitor electron dynamics (and specifically BER) and optical spectroscopy to monitor directly the accumulation and dynamics of surface holes.^17^ In both cases, we employ 5 s-duration light pulses to provide quasi-steady-state irradiation and to enable investigation of the kinetics following light turn on and off.

## Methods

The temperature dependence of the photocatalytic water oxidation reaction was investigated as a function of the applied potential, light intensity, and temperature. We used nanostructured “desert rose” TiO_2_^16,17^ photoanodes previously reported to achieve near-unity quantum efficiencies for water oxidation under strong anodic potential at room temperature.^18,19^ Spectroscopic and photoelectrochemical experiments were performed using a three-electrode cell, as described in Figure S1. Light excitation was provided by a 365 nm light-emitting diode (LED), with intensity varied between 0.11 and 11 mA/cm^2^ (approximately equivalent to 0.01 to 1 sun irradiation), the potential was applied from 0.0 V to +1.6 V vs the reversible hydrogen electrode (*V*_RHE_), and two temperatures, 20 °C and 50 °C, were chosen to represent low and high practical reaction temperatures, respectively. The effect of temperature on the potential versus RHE is disregarded due to a minimal change of 7 mV across the temperature range of 20–50 °C (Figure S2).^20−22^ The pH value was adjusted to 6.9 by using a potassium phosphate (KP) buffer. The absorption of photogenerated TiO_2_ holes was observed at a probe light wavelength of 500 nm, following previous studies.^23−25^ All photocurrent and in situ photoinduced absorption (PIA) measurements are presented as the average of more than four repeated measurements to minimize the potential for experimental error.

## Results

**Figure 1:**
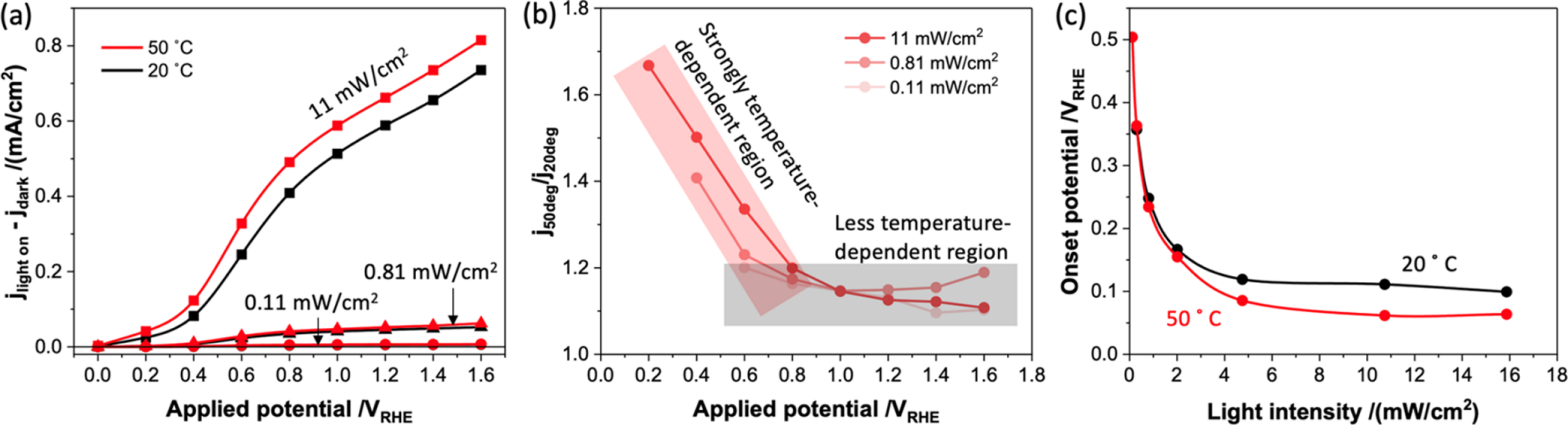
(a) Plot of the relation between the photocurrent and applied potential for nanostructured TiO_2_ photoanodes in a three-electrode cell with aqueous pH 6.9 buffer. Different light intensities (at 355 nm) and temperature data are shown. (b) Plot of the ratio of photocurrent at 50 °C to that at 20 °C determined from the data in (a). (c) Plot of the onset potential versus light intensity at 20 and 50 °C determined from chopped light photocurrent data (see the SI).

Figure 1a shows the relationship between the applied potential and photocurrent at 20 and 50 °C for three light intensities. Under all light intensities and applied potentials, a higher photocurrent was noted at 50 °C than at 20 °C. To quantify the temperature dependence of the photocurrent at each potential, the ratios of photocurrent at 20 and 50 °C are plotted in Figure 1b. The photocurrent increases most significantly with temperature at lower applied voltages. The onset potentials against light intensity at 20 and 50 °C were also compared (Figure 1c, see Figures S3 and S4 and explanation in the Supporting Information (SI) for the determination of this onset potential). The cathodic shift of the onset potential at 50 °C relative to 20 °C is the largest (up to 50 mV) at higher light intensities. We note that these temperature dependencies would be further enhanced if we corrected for the temperature dependence of the RHE. In any case, it is apparent that elevated temperature significantly enhances photocatalytic performance, and this enhancement is mostly pronounced at lower applied voltages. In this lower potential range, kinetic competition can be expected between WOC and BER. As such, we focus our study herein on the temperature dependence of these two competing processes and how these impact the overall photocatalytic performance.

**Figure 2:**
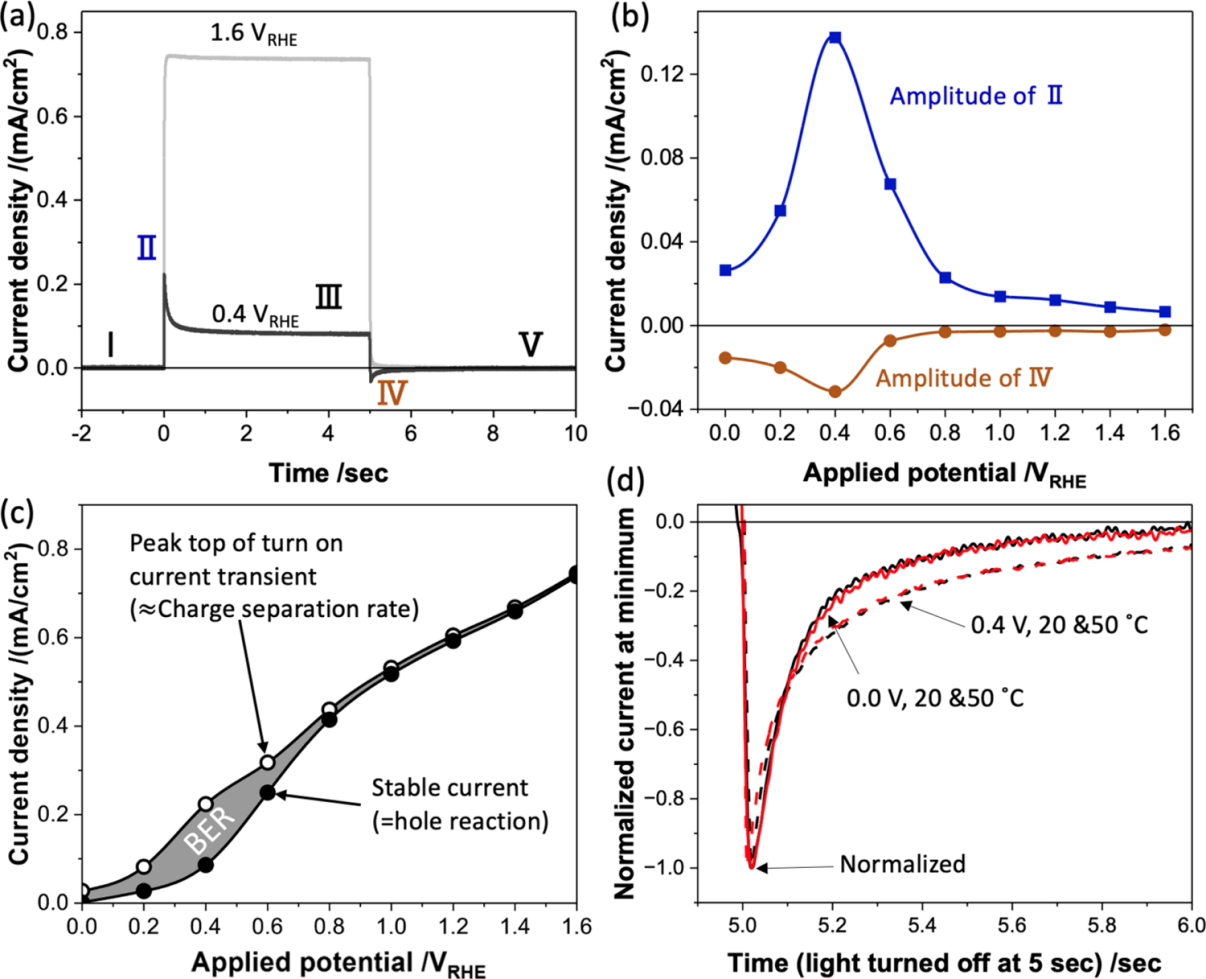
(a) Current transients observed at +0.4 and +1.6 *V*_RHE_ induced by 5 s of light irradiation at 20 °C. (b) Plot of the potential dependence of the amplitude of turn-on/off current spikes marked as II and IV, respectively, in (a). (c) Plot of the top current density following chopped light turn-on (hollow circle) and the stabilized current density (solid circles) as a function of applied potential. Full data are shown in Figure S7. (d) Normalized turn-off (IV) current transients at two low applied potentials (0.0 and +0.4 *V*_RHE_) and two temperatures (20 and 50 °C). All data were obtained for a light intensity of 11 mW/cm^2^. Further experimental details are in Figure 1 caption.

We focus first on the analysis of the turn-on/off current transients observed under chopped light irradiation and how these can be employed to evaluate the kinetics of BER. Typical transients are illustrated in Figure 2a for two different potentials at 20 °C. While almost square wave transients are observed under strong anodic potential (+1.6 *V*_RHE_), turn-on and turn-off current spikes are observed at a lower potential, labeled as II and IV, respectively. The negative current transient observed at light turn-off (IV) has previously been assigned to BER.^10,12−14,26^ This occurs when the potential-induced band bending is insufficient to prevent the recombination of the surface holes with bulk electrons. Figure 2b illustrates the amplitudes of both the turn-on and turn-off spikes relative to the steady current (III) versus the applied potential. The amplitudes of the turn-on and turn-off spikes exhibit similar dependencies on applied potential, both maximal at +0.4 *V*_RHE_. Figure S5 shows that the decay kinetics of the turn-on and turn-off are very similar.^27^ These similarities indicate that the turn-on current transient (II) is also most likely associated with the accumulation of surface holes, which drives BER. Comparing Figure 2b with Figure 1b, the potential region showing the strongest temperature dependence of photocurrent is also the region where the transients assigned to BER are the largest. This impact is further illustrated in Figure 2c, which plots the spike turn-on (II) and steady-state (III) photocurrents against the applied potential. The photocurrent spike can be understood as a measure of the charge separation efficiency η_sep_, while the steady-state photocurrent is the product of charge separation and charge transfer efficiencies (η_sep_ × η_tr_). The deviation between these two plots is an indication of BER losses (we note that this simple analysis neglects band edge unpinning effects; we discuss further below the potential impact of band edge unpinning). It is apparent also from Figure 2c that BER losses are most dominant in the low potential range (<0.8 *V*_RHE_).

We turn now to analyze the temperature dependence of the turn-off transients (IV), in order to examine the temperature dependence of the kinetics of the BER. Figure 2d illustrates these normalized transients at two low applied potentials at 20 and 50 °C. It is striking that the decay kinetics of these transients, assigned to the kinetics of BER, are essentially temperature-independent, with decay half-times of 90 ms in both cases. We also obtained the turn-on current transient (II) at 0.0 *V*_RHE_ for both 20 and 50 °C, showing similar kinetics (Figure S6). This temperature independence is striking and suggests that BER is rate-limited primarily by a tunneling rather than a thermally activated process for the nanostructured TiO_2_ photoanodes studied herein, as will be discussed further below. It also indicates that the strong dependence of the photocurrent upon temperature in this low-potential region does not result from the temperature dependence of BER.

**Figure 3:**
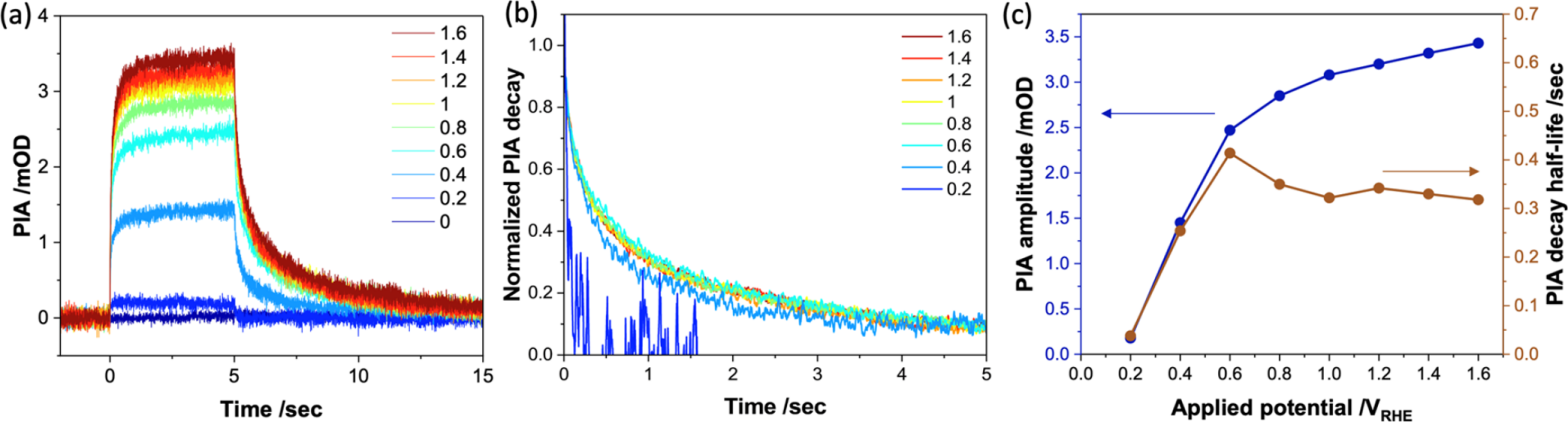
(a) Response of the PIA signal to a 5 s light irradiation. The result was obtained in the same experiment as Figure 2a. (b) PIA decays with different applied potentials normalized with the value at the moment the light turns off. (c) Overlap of the PIA amplitude in (a) and decay half-life in (b) plotted against applied potential. The experiments in these figures were performed at 20 °C, and the light intensity was 11 mW/cm^2^.

We turn now to optical photoinduced absorption (PIA) analysis of the accumulation and decay kinetics of surface TiO_2_ holes generated by the 5 s light pulses.^28^ Such PIA studies have previously focused on light-intensity dependencies under strong anodic potential, where BER is suppressed due to the strong potential. As our focus herein is on the low-potential region where BER and WOC are in kinetic competition, we consider first PIA data as a function of applied potential at a constant light intensity (11 mW/cm^2^ at 365 nm) and temperature (20 °C). Figure 3a displays the resultant PIA transients from 0.0 *V*_RHE_ to +1.6 *V*_RHE_; Figure 3b shows the normalized decay kinetics of these transients. The steady-state PIA amplitudes and decay half-times are plotted against the applied potential in Figure 3c. As the applied potential increases, the PIA amplitude increases, indicating a higher accumulation of surface holes. This can be attributed to increased band bending with a more positive applied potential, which increases η_sep_ and thus the yield of surface holes, as well as suppressing BER, thereby increasing the lifetime of these holes. At high applied potentials of >+0.8 *V*_RHE_, where BER losses are negligible (Figure 2b), the PIA decay kinetics show a modest acceleration with increased potential, assigned to faster WOC with increasing surface hole density.^28^ In contrast, the sharp reduction in PIA decay times at potentials of <+0.6 *V*_RHE_ is assigned to the acceleration and dominance of BER under reduced band bending, consistent with our discussions above. Further support for this conclusion comes from a comparison of the kinetics at +0.4 *V*_RHE_ of the integrated turn-off current spike assigned to BER with the kinetics of the PIA decay at this potential, as plotted in Figure S8. It is apparent that these transients show very similar kinetics, confirming that the acceleration of the PIA transients at low applied potentials results from the surface hole decay kinetics becoming dominated by BER. These data thus clearly illustrate how the kinetic competition between BER and WOC evolves as a function of the applied potential. At high applied potentials, WOC dominates (η_tr_ ∼ 1), while at low potentials, BER suppresses η_tr_.

**Figure 4:**
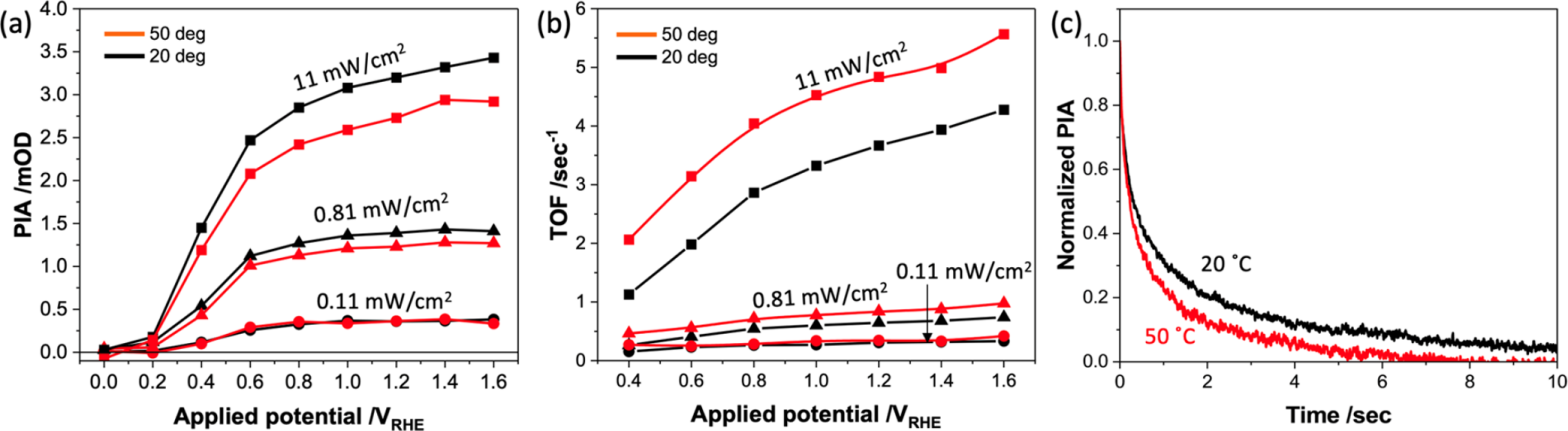
(a) Relation between PIA amplitude and applied potential under different light intensities and temperatures. (b) Turnover frequency (TOF) against applied potential. The TOF values were calculated by dividing the photocurrent by the hole number using previously reported relation.^28^ (c) PIA decays at 20 and 50 °C normalized with the value at the time light turns off after 11 mW/cm^2^ light irradiation. The applied bias for both conditions is 1.6 *V*_RHE_.

We turn finally to the temperature dependence of the PIA transients and the insights they provide into the temperature dependence of the kinetic competition between BER and WOC. Figure 4a displays the amplitude of the stabilized PIA signal as a function of applied potential at 20 and 50 °C and at three light intensities. The amplitude of the PIA signal and, therefore, the density of accumulated surface holes decrease with temperature. This difference is most pronounced at higher light intensities. This reduction in hole accumulation with higher temperature is observed at all potentials and therefore cannot be assigned to faster BER (indeed, as we have discussed above, BER appears to be essentially temperature-independent for the TiO_2_ photoanodes studied herein). Rather, we assign the reduction in hole accumulation to an acceleration of WOC with a higher temperature. Quantification of this acceleration was made by determining the turnover frequency (TOF) of surface holes obtained from dividing the photocurrent densities in Figure 1a by the surface hole densities determined from the PIA data in Figure 4a. This procedure has been reported previously,^29^ with details given in the SI, including eq S1. It should be noted that the key point in this equation is that our analysis is based not on the consideration of reaction site densities, which can be expected to be temperature-independent, but rather on the density of oxidizing species (“holes”) on the surface, which will be dependent on the generation and reaction kinetics of these species and therefore temperature-dependent. The hole TOF increases with applied potential, assigned to the expected acceleration of WOC with increased surface hole density, as discussed above (see also Figure S9). More importantly, it is apparent that this TOF significantly increases at higher temperatures (by up to 2-fold), indicating faster WOC. Figure 4c plots PIA decay kinetics under strong anodic potential at 20 and 50 °C. The decay kinetics are clearly faster at 50 °C, providing further confirmation of faster WOC. Overall, these data indicate that increasing the temperature from 20 to 50 °C results in a 25–70% acceleration (dependent on applied potential and light intensity) of WOC.

Given the temperature independence of BER observed herein and the contrasting temperature dependence of the WOC, our data strongly indicate that the striking enhancement of photocurrent density in the low-potential region with increased temperature (Figure 1b) results from an acceleration of WOC. This acceleration enables water oxidation to compete more effectively with BER and therefore results in the most significant increase in performance in a potential range where kinetic competition between BER and WOC is most significant (i.e., <+0.6 *V*_RHE_).

Further support for the accelerated WOC being the primary cause of the enhanced low-potential region photocurrents at higher temperatures was obtained by tests in the presence of triethanolamine (TEOA) at a fixed temperature of 20 °C. TEOA is known to be a hole scavenger, readily oxidized by TiO_2_ holes, and can therefore be expected to accelerate hole transfer to the electrolyte, equivalent to accelerated catalysis.^30^ In the presence of TEOA, we observed an increase in photocurrent at all potentials (Figure S10), a decrease in PIA amplitudes (Figure S11), and faster PIA decay kinetics (Figures S12 and S13). These effects are all qualitatively analogous to those we report herein with increased temperature (Figures 1 and 4) and support our conclusion that the primary underlying origin of the enhanced photoelectrochemical performance with increased temperature is faster WOC.

**Figure 5:**
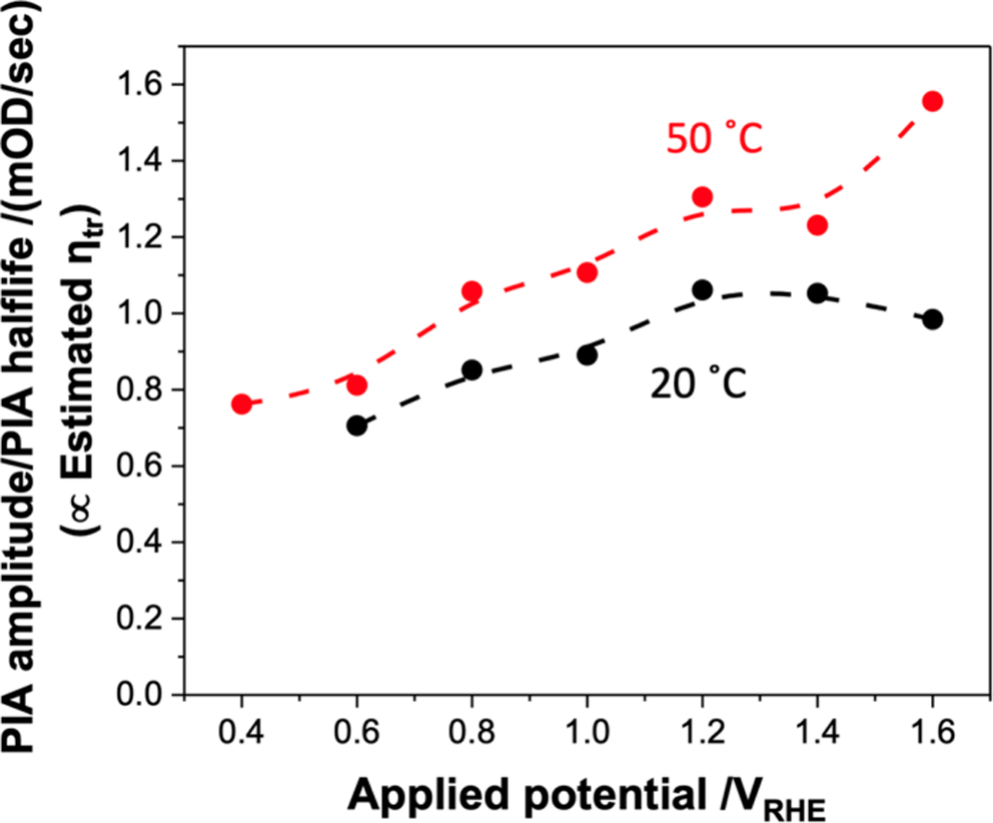
Calculated yield, that is, ratio of PIA amplitude and decay kinetics at 20 and 50 °C, proportional to η_tr_.

We complete our analysis by considering the origin of the smaller, but still significant, photocurrent enhancement observed at higher potentials of >+0.8 *V*_RHE_ (Figure 1b). In this high potential range, BER is strongly suppressed, resulting in a charge transfer efficiency η_tr_ ∼ 1, as we have demonstrated above (see also Figure S15 for an estimate of η_tr_ as a function of applied potential determined from the ratio of the turn-on current spike versus steady-state photocurrent densities in Figure S14). The enhanced photocurrent in this potential range is thus indicative of an enhanced separation efficiency (η_sep_). To quantify this, we utilized the ratio of PIA amplitude to PIA half-life, as this ratio is directly related to the charge separation efficiency. The rationale behind this approach is that under steady-state illumination, the number of photogenerated holes remains constant, ensuring that the generation and consumption of holes are in equilibrium. The PIA amplitude represents the number of surface holes, while the PIA half-life corresponds to the decay rate of these holes. By dividing the amplitude by the half-life, we obtain a value that reflects the rate of hole consumption per unit time, which is proportional to the charge separation efficiency (Figure 5). This analysis confirms a higher yield of surface holes (i.e., higher η_sep_) at 50 °C compared to that at 20 °C. This higher η_sep_ could result from an acceleration of hole transfer to the surface at higher temperature (e.g., a thermally activated hole transport). However, the equivalent analysis at 20 °C with TEOA also shows a higher yield of surface holes (Figure S16) compared with the aqueous electrolyte in the absence of TEOA. Our observation of increased surface hole yield (i.e., η_sep_) with TEOA can be most easily understood by TEOA suppressing band edge unpinning effects.^31^ In the absence of TEOA, the higher accumulated hole densities can be expected to result in great band edge unpinning (i.e., suppressed band bending), which would lower η_sep_. Given the analogous increase in surface hole yield we observe at 50 °C in the aqueous electrolyte, it appears most likely that the modest increase in photocurrent observed with increased temperature in the high applied potential range also results, at least in part, from suppressed band edge unpinning. This most likely primarily results from faster WOC lowering the steady-state density of accumulated surface holes.

## Discussion

Our results herein highlight that raising the reaction temperature from 20 to 50 °C can significantly enhance the water oxidation efficiency of nanostructured titania photoanodes. The temperature increase from 20 to 50 °C during photocatalytic reactions is realistic considering the typical daily, annual, and geographical temperature variations. The relative performance enhancement at 50 °C is the greatest (up to 70% higher photocurrent) in the lower applied potential regime up to +0.6 *V*_RHE_. This lower potential regime is particularly critical for the performance in photocatalytic systems such as powders and films that are not subjected to an electric potential.^2,32^ This enhancement is assigned primarily to elevated temperature, increasing the charge transfer efficiency, η_tr_, determined by kinetic competition between WOC and BER (also called surface recombination). In particular, this enhancement results from the BER kinetics being temperature-independent, while the WOC kinetics accelerate by up to 2-fold. At more anodic applied potential, a much smaller (10%) enhancement in photocurrent is observed, assigned to a modest increase in the charge separation efficiency, η_sep_.

In Figure 1b, it is evident that the temperature dependence is more pronounced at higher light intensities. This phenomenon can be explained by two possibilities. One possibility is that the WOC becomes more temperature-dependent at higher light intensities. According to our previous works on rate law analysis, the number of holes involved in the reaction changes in accordance with the surface hole density,^17,28,33^ which suggests a corresponding temperature dependence in the kinetics. This could explain why WOC appears to be especially advantageous over BER at higher light intensities. A second possibility is that band edge unpinning plays an important role. Since unpinning is pronounced when the hole density is high, the unpinning effect is minimal at low light intensities, regardless of the rate of WOC. On the other hand, under high light intensities, the unpinning effect becomes prominent at low temperatures due to slow WOC and high hole density. However, as the temperature increases and WOC accelerates, the hole density decreases, making the unpinning effect less significant. Consequently, band bending becomes steeper with increasing temperature, allowing the spatial separation of surface holes and excited electrons, which could suppress BER. In any case, at high light intensities, WOC becomes dominant over BER at high temperatures, causing a clearer temperature dependence, as Figure 1c demonstrates.

The acceleration of WOC with elevated temperature is indicative of an activation energy for this reaction on the order of 150–200 meV at 0.4 *V*_RHE_. This acceleration is observed across all applied potentials but impacts the overall WOC efficiency most significantly at low applied potentials where it is in kinetic competition with BER. We note that we have previously analyzed in detail the activation energy for water oxidation on hematite photoanodes and observed this to decrease from ∼300 meV at low surface hole coverages to 60 meV at high hole coverages (>1 hole/nm^2^).^29^ The PIA optical signals observed herein are indicative of surface hole coverages on our nanostructured titania of up to 9.4 holes/nm^2^.^28^ We also note that transient absorption studies have previously suggested a lower activation energy for titania nanoparticles.^23^ Further study at a broader range of temperatures and samples is necessary to investigate this point further.

BER is based on the recombination of surface holes with bulk electrons. There are two possible mechanisms for this reaction. One would be expected to be thermally activated by the band bending present across the space charge layer (e.g., surface holes diffusing against this barrier back into the hematite bulk and recombining with bulk electrons). The other corresponds to direct tunneling recombination across the space charge layer, which would be expected to be relatively temperature-independent. Our observation of temperature-independent BER kinetics indicates the dominance of the tunneling pathway. The titania films studied herein have an electron carrier density of 6 × 10^18^ [cm^–3^],^18^ yielding a space charge layer width of ∼30 nm at 0.4 *V*_RHE_ (eq S2 in the SI).^28^ It is striking that despite this width, this tunneling BER pathway dominates, perhaps mediated via intragap states such as oxygen vacancies or by inhomogeneities in space charge layer thickness.

## Conclusions

Our study herein has unraveled how the temperature dependencies of the underlying kinetic processes combine to determine the temperature dependence of the overall photocatalytic efficiency in nanostructured titania photoanodes. It is likely that the temperature dependencies of these kinetics will vary between photoanode materials, due to differences in factors such as the activation energy for water oxidation catalysis or the impact of doping density on the temperature dependence of back electron–hole recombination. For example, we note that a very recent study has reported thermally activated BER on flat TiO_2_ photoanodes, most likely due to differences in morphology or doping density relative to the photoanodes studied herein.^34^ Elucidating and controlling these temperature dependencies will be crucial for optimizing practical device operation, given the varying operating temperatures of practical photoreactors.

## Supporting Information Available

Equations for turnover frequency and space charge layer width, list of chemicals and materials used, preparation of TiO_2_ photoanodes, schematic view of the experimental setup, temperature dependence of reference potential, determination of onset potential through chopped light linear sweep voltammetry, turn-on/off current transients, current density–voltage curves with and without triethanolamine (TEOA) in the aqueous electrolyte, PIA signal decay and half-time with and without TEOA, photocurrent density, η_tr_, and yield calculations as functions of applied potential and experimental conditions (PDF)
