# Supplementary material for: Analyzing the Temperature Dependence of Titania Photocatalysis: Kinetic Competition between Water Oxidation Catalysis and Back Electron–Hole Recombination
Source: ACS Catal. 2024 Oct 24;14(21):16543–50. doi: 10.1021/acscatal.4c03685 (PMC11537271; doi:10.1021/acscatal.4c03685)
Supplement: Supplementary file 1 — cs4c03685_si_001.pdf [file cs4c03685_si_001.pdf]

Supporting Information
Analysing the Temperature Dependence of Titania Photocatalysis: Kinetic Competition between Water Oxidation Catalysis and Back Electron-Hole Recombination

Authors: *Yohei Cho[1-3], Tianhao He[1], Benjamin Moss[1], Daniele Benetti[1], Caiwu Liang[1], Lei Tian[1], Lucy Jessica F Hart[1], Anna A. Wilson[1], Yu Taniguchi[2], Junyi Cui[4], Mengya Yang[4], Salvador Eslava[4], Akira Yamaguchi[2], Masahiro Miyauchi[2], and James R Durrant[1*]*

AUTHOR ADDRESS:

1 Department of Chemistry and Centre for Processable Electronics, Imperial College London, London W12 0BZ, United Kingdom

2 Department of Materials Science and Engineering, School of Materials and Chemical Technology, Tokyo Institute of Technology, 2-12-1 Ookayama, Meguro-ku, Tokyo 152-8552, Japan

3 Graduate School of Advanced Science and Technology, Japan Advanced Institute of Science and Technology, 1-1 Asahidai, Nomi, Ishikawa 923-1292, Japan

4 Department of Chemical Engineering and Centre for Processable Electronics, Imperial College London, London SW7 2AZ, United Kingdom

Corresponding Author

* E-mail: j.durrant@imperial.ac.uk

Equation S1:

$$\mathrm{TOF[sec^{-1}]} = \frac{j\left[\frac{\mathrm{mA}}{\mathrm{cm^2}}\right] \times \frac{1}{9.65\times 10^4\left[\frac{\mathrm{C}}{\mathrm{mol}}\right]} \times 10^{-3}}{\mathrm{PIA[m\Delta OD]} \times \frac{5.2\times 10^{-8}\left[\frac{\mathrm{mol\cdot cm}}{\mathrm{L}}\right] \times 10^{-3}\left[\frac{\mathrm{L}}{\mathrm{cm^3}}\right]}{0.1\ \mathrm{[m\Delta OD]}}}$$

The value 5.2×10^{-8} [mol cm L^{-1}] and 0.1 mOD were obtained from a previous report.[1]

Equation S2:

$$W\mathrm{[cm]} = \left(\frac{2\varepsilon_{0\mathrm{[Fcm^{-1}]}}\varepsilon_r}{q\mathrm{[C]}N_D\mathrm{[cm^{-3}]}}\right)^{1/2}\left(V\mathrm{[V]} - V_{fb}\mathrm{[V]} - \frac{k\mathrm{[JK^{-1}]}T\mathrm{[K]}}{q\mathrm{[C]}}\right)^{1/2}$$

The width of space charge layer was calculated using Equation 2. Here, the flat band potential $V_{fb} = 0.12\ [\mathrm{V_{RHE}}]$ was used following previous report.[2]

Materials

Titanium(iv) ethoxide $Ti(OEt)_4$, anhydrous toluene (≥99.9%) and ethanol (<0.0003% water) were provided by Sigma Aldrich. Aluminoborosilicate glass (ABS) coated with a fluorine-doped tin oxide (FTO) transparent conductive layer (8 Ω sq^{-1}) was provided by Solaronix SA, Switzerland. This FTO-ABS was cleaned by ultrasonication in a 2% aqueous Hellmanex III solution, deionized water, acetone and isopropyl alcohol (each step for 3 min), followed by rinsing in deionized water and dried.

Preparation of TiO_2 photoanodes

TiO_2 photoanodes on ABS-FTO substrates were prepared by aerosol-assisted chemical vapor deposition (AACVD) as previously published elsewhere by Regue et al.[2] First, $Ti_7O_4(OEt)_{20}$ clusters were prepared by controlled hydrolysis of $Ti(OEt)_4$ in toluene at room temperature.[3] Next, these clusters were dissolved in toluene at a concentration of 0.05 M and placed in a TSI Model 3076 Constant Output Atomiser for aerosol generation using and nitrogen as a carrier gas at a constant flow rate of 1.5 L min^{-1}. AACVD of $Ti_7O_4(OEt)_{20}$ was carried out onto FTO–ABS substrates placed horizontally inside a tube furnace of 34 mm diameter. Deposition time was 1 h and tube temperature 500 °C. At the end of the deposition, the substrate was left to cool down under nitrogen flow. The obtained films were further annealed in air at a heating rate of 10 °C min^{-1} up to 800 °C, kept at this temperature for 2 h, and then left to cool down in air. The resultant TiO_2 had a thickness of 2.5 μm with {101} facet preferentially exposed.[2]

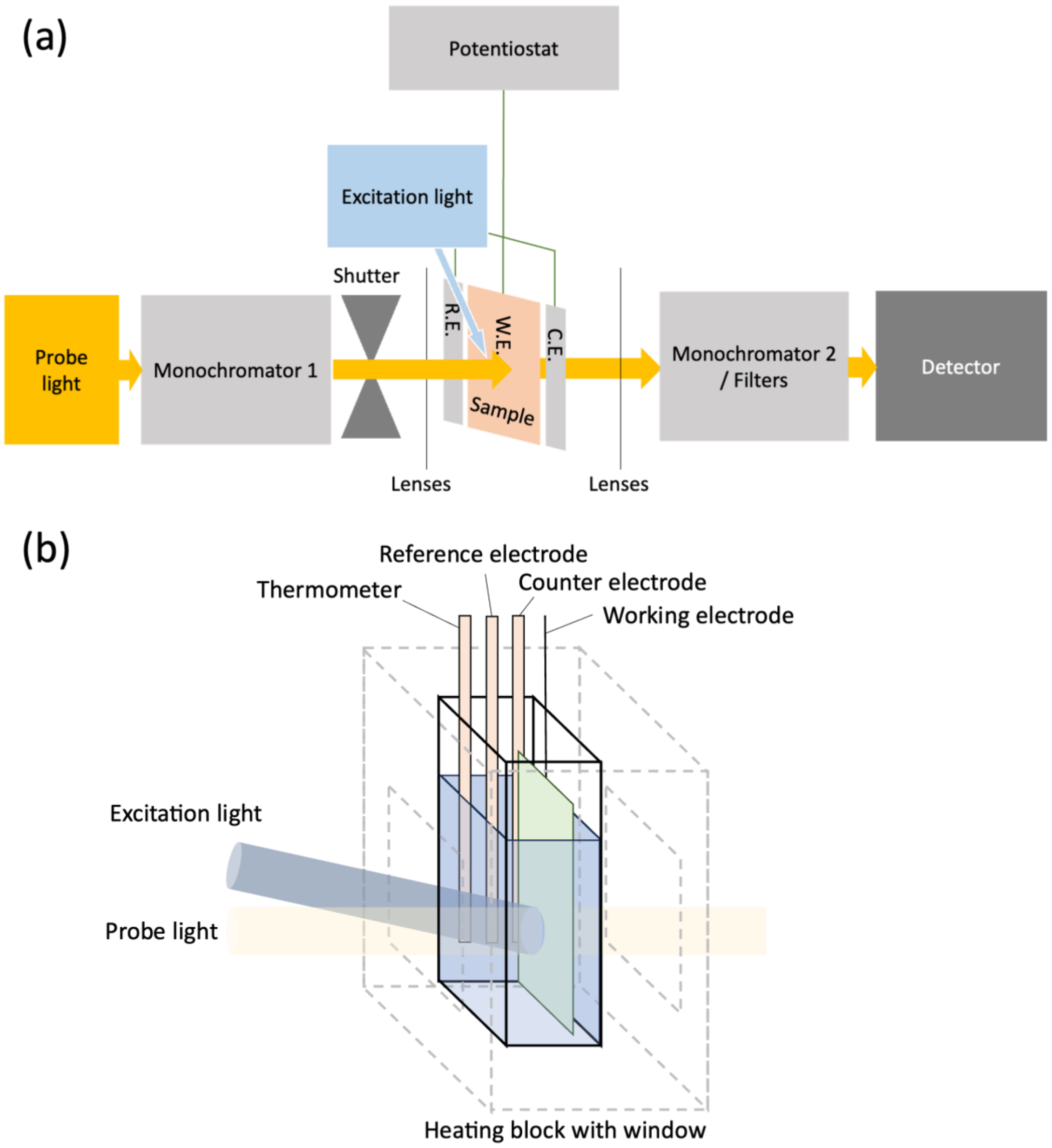

Figure S1(a) Schematic view of the experimental setup. Continuous light at 500 nm is irradiated through monochromators onto the working electrode and then directed to the detector. The excitation light is applied for 5 seconds, during which transient current and absorption are measured by the potentiostat and photodetector, respectively. **(b)** Detailed configuration around the photoelectrochemical reactor. A typical three-electrode system is employed, with a thermometer detecting the solution temperature. The temperature feedback is used to control the heating block.

Temperature dependence of potential

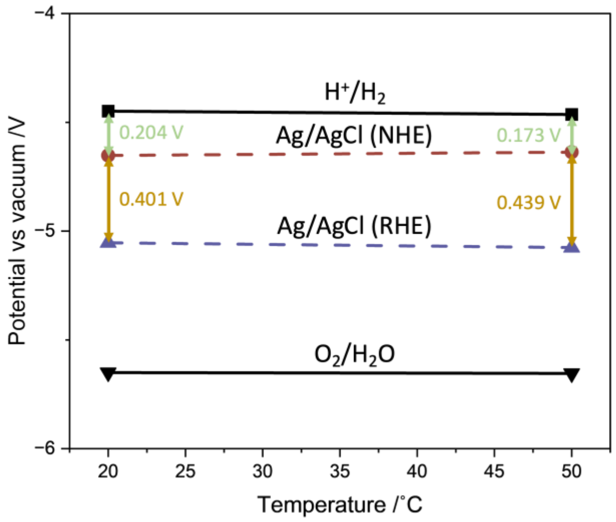

Figure S2 Tempearture dependence of each potential versus vacuum. The temperature dependent value of $E_{\mathrm{H^+/H_2}}$, $E_{\mathrm{Ag/AgCl}}$, and pH refered to previous works.[4–6]

The potential values were calculated based on the following equation:

$$E_{\mathrm{Ag/AgCl\,,vs\,vac}} = E_{\mathrm{H^+/H_2}} - E_{\mathrm{Ag/AgCl}} - \frac{RT}{zF}\mathrm{pH}$$

$$E_{\mathrm{Ag/AgCl(RHE),\,20,vs\,vac}} = -4.448 - 0.204 - \frac{2.3026 \times 8.31 \times 293}{1 \times 9.65 \times 10^4} \times 6.90$$

$$E_{\mathrm{Ag/AgCl(RHE),50,vs\,vac}} = -4.464 - 0.173 - \frac{2.3026 \times 8.31 \times 323}{1 \times 9.65 \times 10^4} \times 6.85$$

Thus,

$$E_{20,RHE} = 0.605\ [V_{RHE}]$$
$$E_{50,RHE} = 0.612\ [V_{RHE}]$$

The difference in values is negligible, only 7 mV.

Determination of onset potential

The onset potential shown in Figure 1(c) was determined using the following method.

1. Fit the light-on and dark lines from chopped light linear sweep voltammetry result.

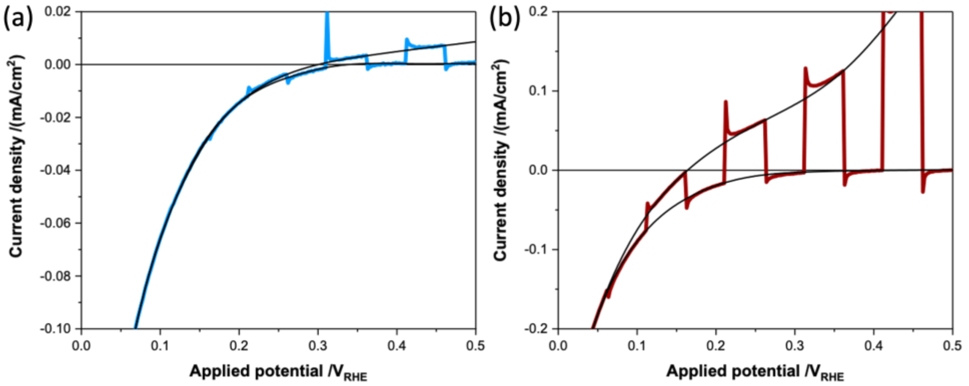

Figure S3 The chopped linear sweep voltammetry result and fitted lines under (a) 0.3 mW/cm^2 light irradiation at 50 ˚C, and under (b) 15.9 mW/cm^2 at 50 deg, shown as examples.

2. Subtract the dark current density from light on current density.

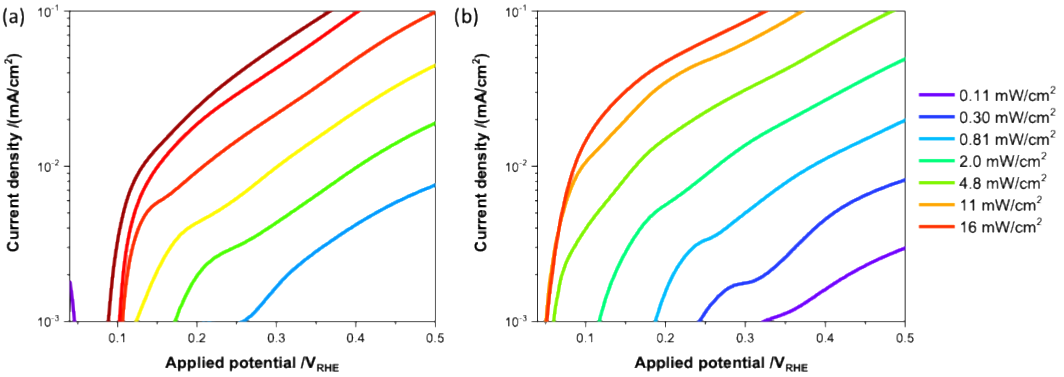

Figure S4 Current density under illumination with dark current subtracted at (a) 20 and (b) 50 ˚C.

3. Read the potential reaching 1uA/cm^2, as it is the detection limit, and obtained the onset potential.

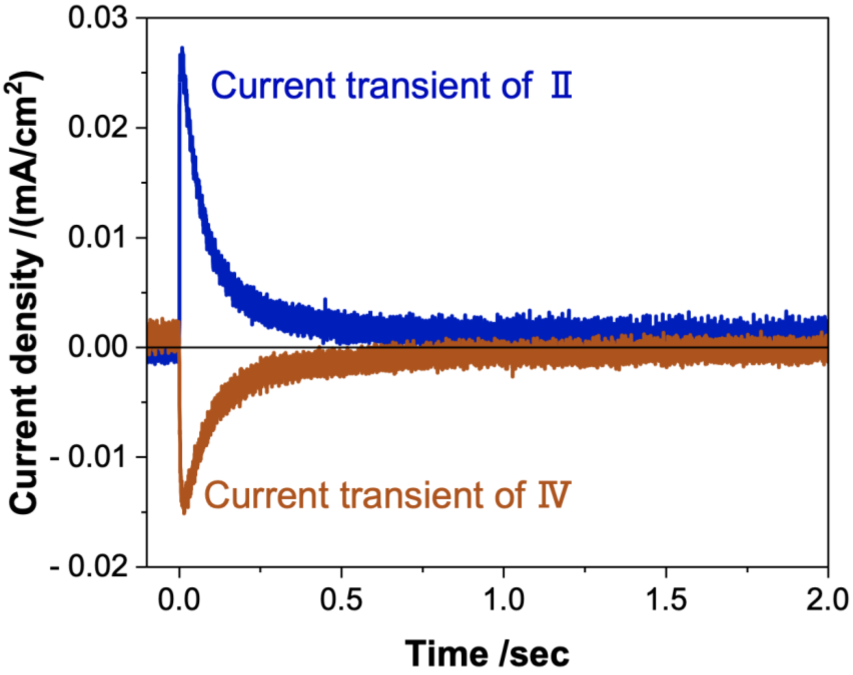

Figure S5 Turn-on (II) and off (IV) current transients at 0.0 V_{RHE}.

We note this increase in BER flux with surface hole density may result in part from a reduction in band bending with increasing hole density (band edge unpinning), although the likely charge neutrality of surface TiO_2 due to proton release is likely to minimize this.

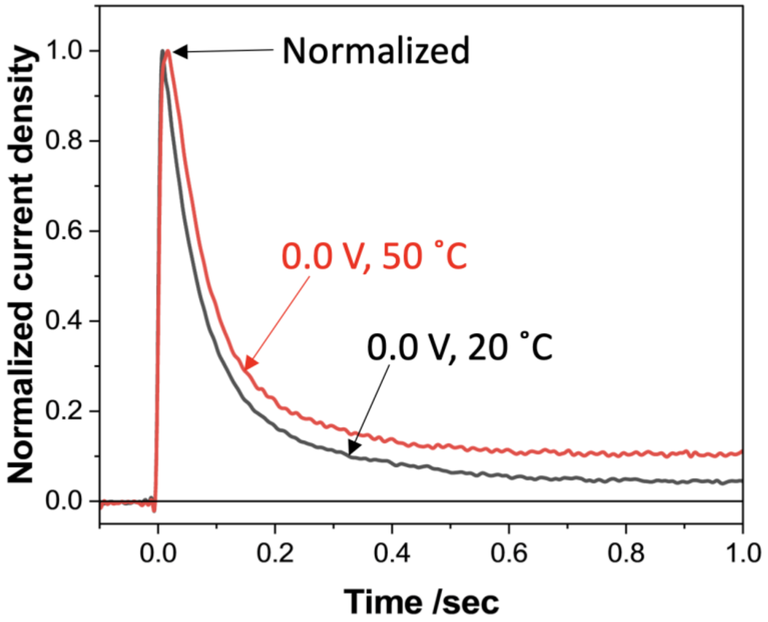

Figure S6 Normalised turn-on (II) current transient at 0.0 V_{RHE}. Light intensity is 11 mW/cm^2.

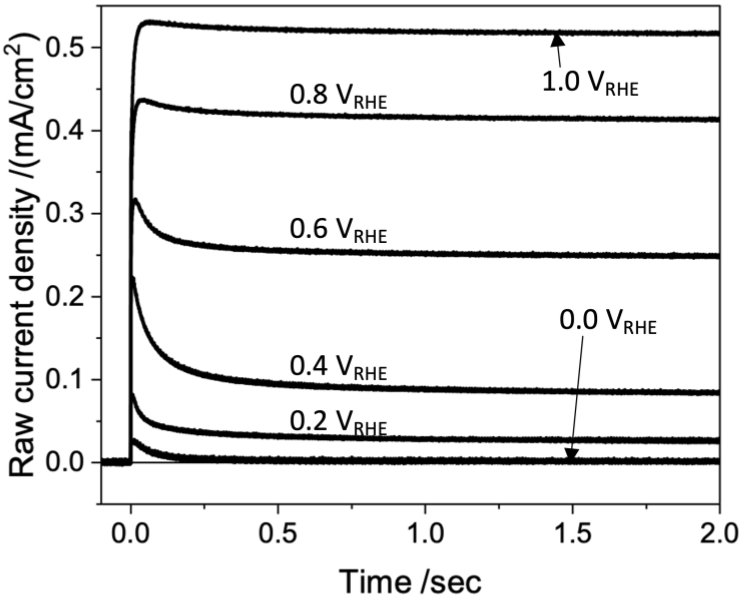

Figure S7 Current transients observed after light turning on at 0 s. The light intensity is 11 mW/cm^2, the temperature is 20 ˚C.

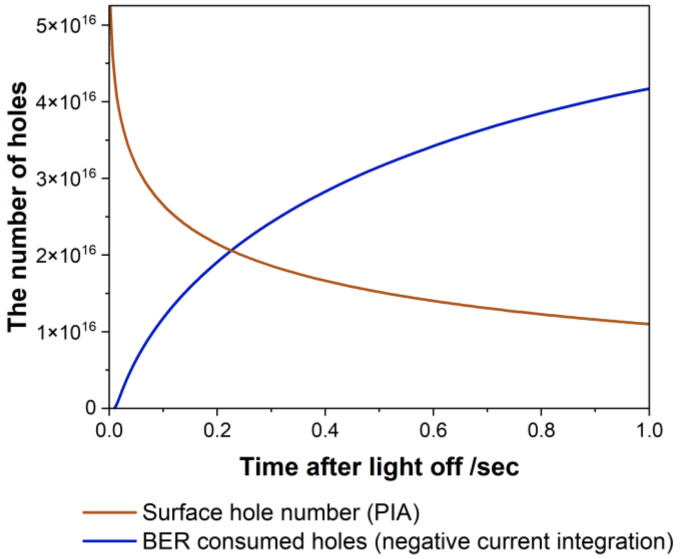

Figure S8 Comparison between the hole number decay calculated from PIA using extinction coefficient,[1] and the holes number consumed by BER process calculated by integrating the negative current observed after light turn off. The experiment was conducted at 0.4 V_{RHE}, at 20 ˚C, under 11 mW/cm^2 light irradiation.

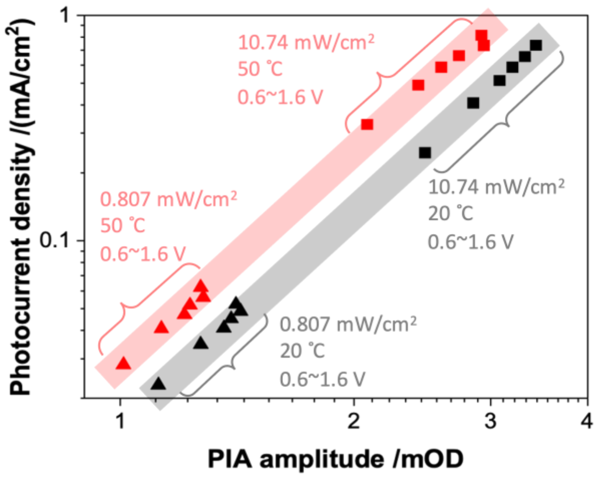

Figure S9 Relationship between photocurrent density and surface accumulated holes. The light intensities, applied biases, and reaction temperature are specified in the figure.

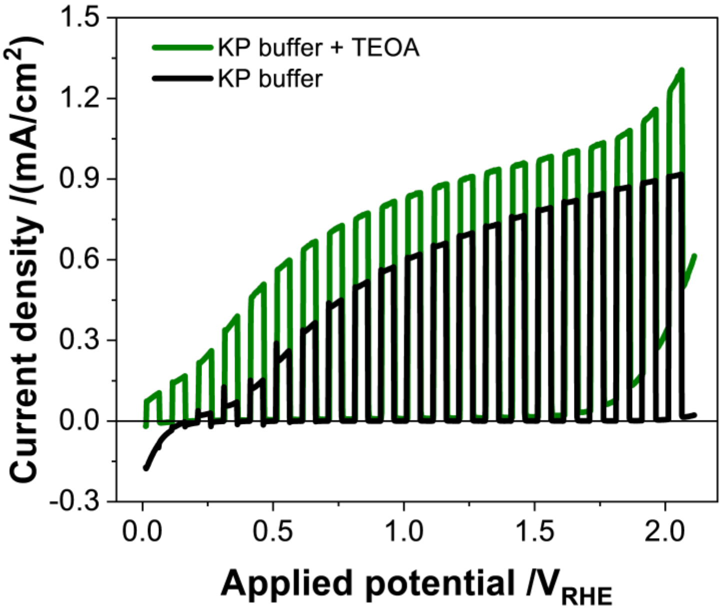

Figure S10 Photocurrent density-voltage curves with chopped illumination with(green) and without (black) triethanolamine (TEOA) in the aqueous electrolyte.The light intensity is 11 mW/cm^2.

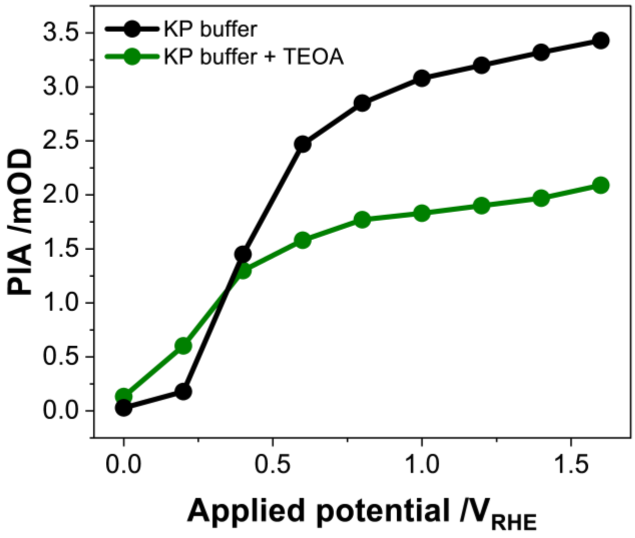

Figure S11 PIA amplitude with TEOA (dark green) and without TEOA (black) in the aqueous electrolyte.

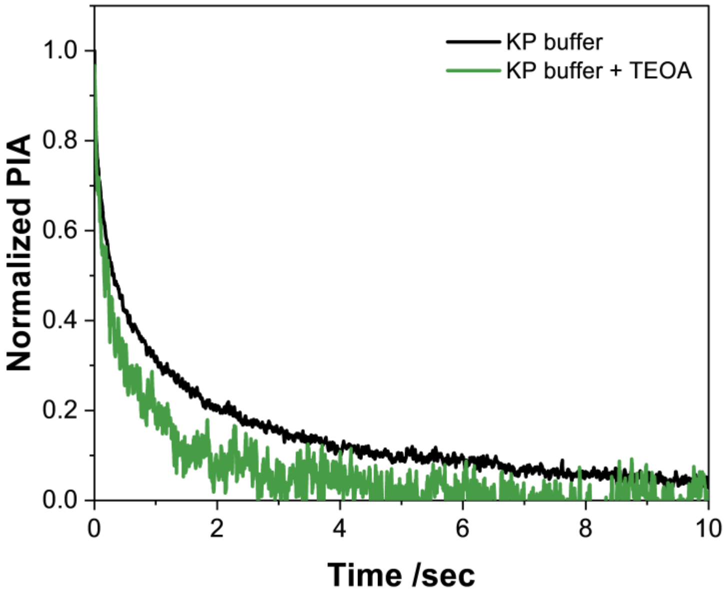

Figure S12 PIA signal decay after steady light irradiation (+1.6 V_{RHE}, 11 mW/cm^2) without TEOA (black line) and with TEOA (dark green line).

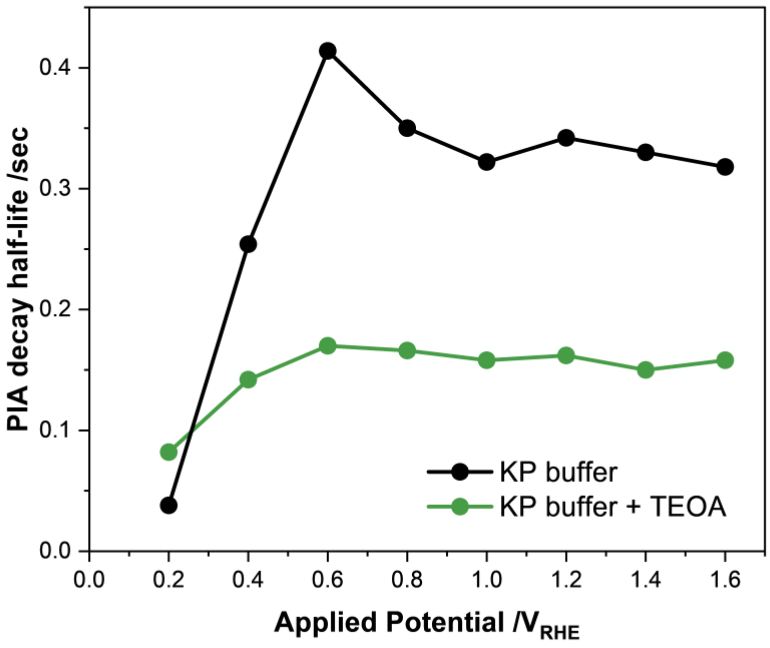

Figure S13 PIA decay half-time with (green) and without (black) TEOA.

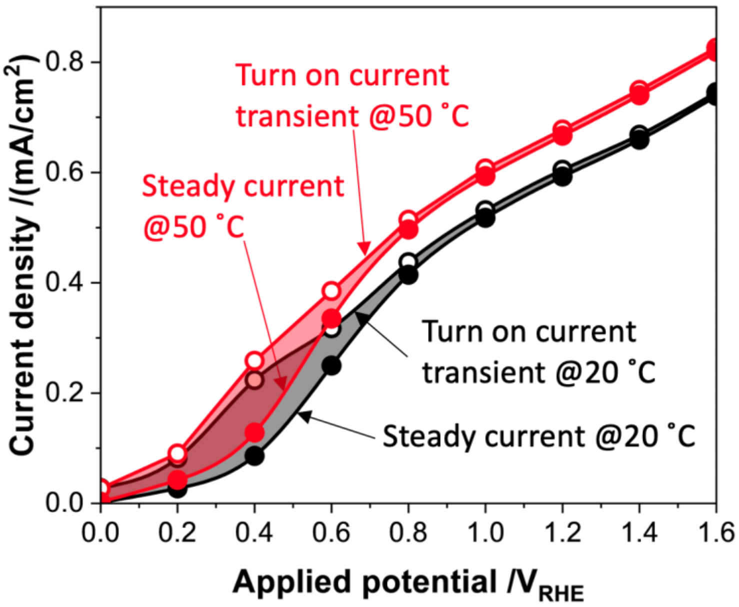

Figure S14 Plot of Figure 2(c) updated with the addition of data at 50°C, showing the peak current density following chopped light turn-on (hollow circles) and the stabilized current density (circles) as a function of applied potential.

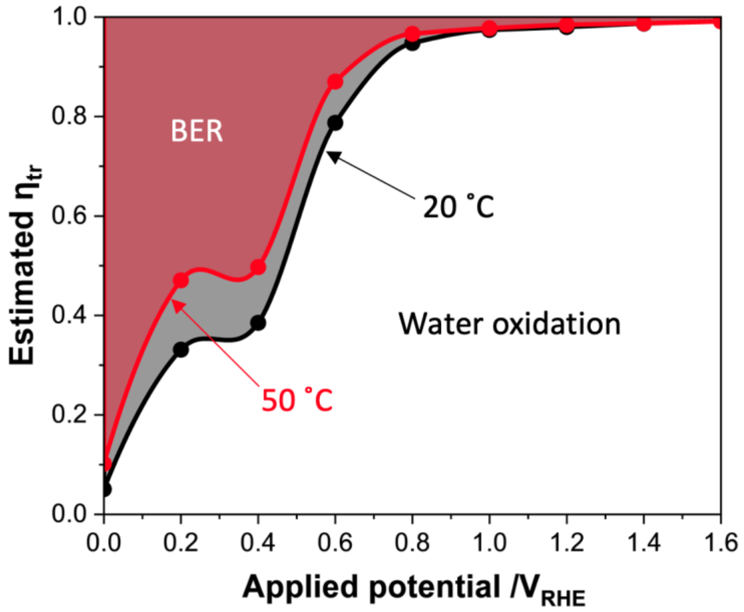

Figure S15 Estimated η_{tr}, calculated by dividing the steady current by the turn on current transient, both in Figure S14.

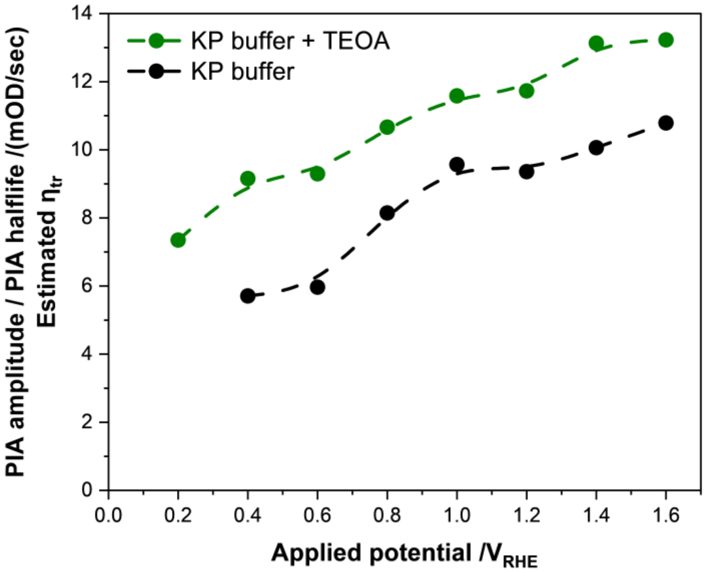

Figure S16 Calculated yield, that is ratio of PIA amplitude and decay kinetics (Eq.1) for experimental conditions with and without TEOA.

References

(1) Kafizas, A.; Ma, Y.; Pastor, E.; Pendlebury, S. R.; Mesa, C.; Francàs, L.; Le Formal, F.; Noor, N.; Ling, M.; Sotelo-Vazquez, C.; Carmalt, C. J.; Parkin, I. P.; Durrant, J. R. Water Oxidation Kinetics of Accumulated Holes on the Surface of a TiO2 Photoanode: A Rate Law Analysis. *ACS Catal* **2017**, *7* (7), 4896–4903. https://doi.org/10.1021/acscatal.7b01150.

(2) Regue, M.; Sibby, S.; Ahmet, I. Y.; Friedrich, D.; Abdi, F. F.; Johnson, A. L.; Eslava, S. TiO2 Photoanodes with Exposed {0 1 0} Facets Grown by Aerosol-Assisted Chemical Vapor Deposition of a Titanium Oxo/Alkoxy Cluster. *J Mater Chem A Mater* **2019**, *7* (32), 19161–19172. https://doi.org/10.1039/c9ta04482e.

(3) Eslava, S.; P. R. Goodwill, B.; McPartlin, M.; S. Wright, D. Extending the Family of Titanium Heterometallic–Oxo–Alkoxy Cages. *Inorg Chem* **2011**, *50* (12), 5655–5662. https://doi.org/10.1021/ic200350j.

(4) Ma, Z.; Liu, W.; Yang, W.; Li, W.; Han, B. Temperature Effects on Redox Potentials and Implications to Semiconductor Photocatalysis. *Fuel* **2021**, *286*, 119490. https://doi.org/https://doi.org/10.1016/j.fuel.2020.119490.

(5) Masamura, K. Reference Electrode. *Boshoku Gijutsu* **1986**, *35*, 422–423.

(6) *Buffers. A Guide for the Preparation and Use of Buffers in Biological Systems*, 3rd ed.; Chandra, M., Ed.; EMD Bioscience, 2006.
